# Exploring the ethics of global health research priority-setting

**DOI:** 10.1186/s12910-018-0333-y

**Published:** 2018-12-06

**Authors:** Bridget Pratt, Mark Sheehan, Nicola Barsdorf, Adnan A. Hyder

**Affiliations:** 1 Nossal Institute for Global Health and Centre for Health Equity, School of Population and Global Health, University of Melbourne, 207 Bouverie St Street, Carlton, VIC 3053 Australia; 2 Ethox Centre, Nuffield Department of Population Health, University of Oxford, Oxford, UK; 3 Health Research Ethics, Faculty of Medicine and Health Sciences, Stellenbosch University, Stellenbosch, South Africa; 4 Milken Institute School of Public Health, George Washington University, Washington, USA

**Keywords:** Ethics, Global health research, Priority-setting, Justice

## Abstract

**Background:**

Thus far, little work in bioethics has specifically focused on global health research priority-setting. Yet features of global health research priority-setting raise ethical considerations and concerns related to health justice. For example, such processes are often exclusively disease-driven, meaning they rely heavily on burden of disease considerations. They, therefore, tend to undervalue non-biomedical research topics, which have been identified as essential to helping reduce health disparities. In recognition of these ethical concerns and the limited scholarship and dialogue addressing them, we convened an international workshop in September 2015. The workshop aimed to initiate discussion on the appropriate relationship between global and national levels of health research priority-setting and to begin exploring what might be ethically required for priority-setting at each of those levels.

**Main text:**

This paper comprises our reflections following the workshop. Its main objective is to launch a research agenda for the ethics of global health research priority-setting. We identify three domains of global health research priority-setting—scope, underlying values and substantive requirements, and procedural considerations. For each domain, specific research questions are highlighted and why they need to be explored is explained. Some preliminary thoughts and normative arguments as to how the research questions might be answered are also offered. For example, we provide initial ideas about the appropriate relationship between different priority-setting levels and what values and substantive considerations should guide or underpin global health research priority-setting as a matter of justice.

**Conclusion:**

We anticipate that framing a new research agenda for the ethics of global health research priority-setting will spur ethicists, researchers, and policymakers to refocus their efforts on developing more rigorous and ethically sound approaches to priority-setting.

## Background

Research ethics has traditionally focused on issues that arise within the researcher-participant relationship such as ensuring informed consent, a favourable risk-benefit ratio, and fair subject selection. In recognition of the growing global context of research and lack of benefits accruing to vulnerable populations, particularly in low and middle-income countries (LMICs), Benatar and Singer ([5], p. 826) argued “a new, proactive research ethics... must ultimately be concerned with reducing inequities in global health and achieving justice in health research and healthcare.” This call to expand the scope of research ethics has been reiterated by others [19, 22, 34]. It is also consistent with calls to expand the more general bioethics agenda to focus on population health and health disparities between and within countries [12, 23].

When the scope of research ethics encompasses efforts to promote health justice domestically and globally, the targets of ethical analysis expand to include health research priority-setting and resource allocation, research translation, and research capacity strengthening [6, 34]. In this paper, we focus on the first of those targets—*priority-setting in research*, particularly in relation to global health research. We define global health research as research addressing health problems worldwide, including those of the most disadvantaged, who live primarily (but not exclusively) in LMICs [24, 31].

Decisions about what global health research is prioritised matter to the achievement of health justice. While largely setting aside ongoing debates about the scope and meaning of health justice, as they are not the focus of our paper, we adopt a middle-ground position: to achieve health justice is to bring individuals worldwide up to at least a decent level of health, with some priority going to those who are worst-off or disadvantaged.^1^ Achieving health justice requires the provision of public health and healthcare goods and services, establishing institutional structures for health, and having broader social arrangements (political, legal, economic, and cultural) that are organized to promote and sustain individuals’ health [13, 39, 44].

Yet whether individuals worldwide, including the vulnerable and disadvantaged, receive preventative care and treatment for their illnesses in part depends on what public health interventions and what medicines have been developed and whether health systems can deliver them efficiently and affordably. This, in turn, depends on what global health research has been prioritised and performed. Where global health research does not focus on particular health conditions (e.g. neglected tropical diseases), effective public health interventions and medicines will not be developed for them. Where global health research does not generate new knowledge on strategies for those interventions and medicines’ delivery and financing, health systems will not be optimised to promote population health and to reduce health disparities. As a result, some individuals, especially in LMICs, will be more likely to get preventable illnesses and to lack access to effective treatments for them.

Despite significant scholarship on research ethics and the ethics of healthcare resource allocation, limited work in bioethics has specifically focused on health research priority-setting. That existing work has largely concentrated on the lack of resources allocated to research on health problems primarily experienced in LMICs. In 1990, the Commission on Health Research for Development’s landmark report, *Health research: Essential link to equity in development* estimated that only 5% of the world’s resources for health research were being applied to the health problems of LMICs, where 93% of the world’s preventable deaths occurred [10]. The disparity subsequently became known as the 10/90 gap and there has been much consideration about how it should be addressed by philosophers, bioethicists, policymakers, and organisations such as the Council on Health Research for Development and the World Health Organization [28, 32, 46]. There continues to be significant effort globally, often led from the global south, to push for decision-making for research to move to LMICs and to support capacity strengthening initiatives aimed at promoting that shift.

Beyond the still relevant 10/90 gap, we draw attention to *three* aspects of global health research priority-setting that raise ethical considerations and concerns connected to health justice. First, priority-setting occurs at different levels, with global priorities often influencing and possibly determining national priorities for health research in LMICs. It has been argued that states—rather than global actors—should be primarily responsible for ensuring their population’s health and, in effect, setting their national research priorities [35]. The complex and varied nature of health systems between countries means local stakeholders are often better situated to identify national research priorities that advance population health and/or contribute to the reduction of health disparities in their countries [7]. This raises a key ethical question: what should the appropriate relationship between different levels of research priority-setting comprise? Should national priorities, for example, direct global priorities or vice versa? A lack of ethical debate and guidance exists on this topic.

Second, there is also a lack of discussion and consensus in the bioethics literature on what values ought to be reflected and what associated substantive requirements ought to be used in global health research priority-setting. Nuyens ([29], p. 320) affirms that health research priority-setting is a “value-driven and political activity” and should, in particular, be driven by the aim of advancing health equity. This is consistent with our position that global health research priority-setting has an essential role to play in promoting health justice and with recommendations made at global ministerial summits that health research should help reduce health disparities between and within countries [26]. However, no specific substantive requirements for equity-oriented health research priority-setting have been described to guide policymakers [36]. There are also other values and substantive requirements related to them that should be included in deliberations about global health research priority-setting. What they are and how they should be reflected/implemented in priority-setting processes requires further investigation.

Third, also of ethical concern are the *processes* through which global health research priorities are set. At the global level, such processes have tended to be top-down, reliant on a limited consultative process, and unable to consider fine-grained local issues [2]. It has been suggested that global health justice demands a system of shared health governance, under which global priorities reflect the true consensus of global, national, *and* sub-national actors [35]. Additionally, global health research priority-setting processes are often exclusively disease-driven, meaning they rely heavily on burden of disease considerations. Such processes tend to undervalue non-biomedical research, resulting in the identification of fewer health systems research priorities and more basic science and clinical research priorities [37]. This is concerning from a health justice perspective because implementation research, health systems research, and research on broader social determinants of health are required to address the health needs of individuals worldwide, particularly the vulnerable and disadvantaged [4, 47, 48]. Another key question to explore is then: what is required for fair and just global health research priority-setting processes?

In recognition of these ethical concerns and the limited scholarship and dialogue addressing them, we convened an international workshop of health researchers, research managers, philosophers, lawyers, bioethicists and ethics review committee members in September 2015. The workshop aimed to initiate discussion on the relationship between global and national levels of priority-setting and to start thinking about what might be ethically required for global health research priority-setting at each of these levels. The workshop aims were then primarily exploratory. Given the lack of thinking and work on the ethics of global health research priority-setting, the intent was to articulate and discuss initial ideas on the two topics. No attempt was made to generate a consensus position based on the workshop discussions. Workshop speakers and participants spanned five continents and twelve countries: Botswana, Uganda, India, South Africa, Ghana, Australia, Canada, Pakistan, UK, USA, Zimbabwe and Zambia. The structure of the workshop was a mix of formal presentations and extensive group interactions; their content informed this paper. We indicate throughout the paper where particular ideas came from workshop speakers or participants and where broad (though generally not overall) support amongst workshop attendees existed for certain ideas. However, we emphasise that the paper is not a product of the workshop but rather a set of reflections arising out of it. It describes our thinking on global health research priority-setting in light of the workshop’s presentation and discussion content.

The main aim of the paper is to launch a research agenda for the ethics of global health research priority-setting. We identify three domains of research priority-setting—scope, underlying values and substantive requirements, and procedural considerations. For each domain, we highlight specific research questions and explain why they need to be explored. We also offer some preliminary thoughts and normative arguments on how the research questions might be answered. For example, we provide initial ideas about the appropriate relationship between different priority-setting levels and what values and substantive considerations should guide or underpin global health research priority-setting as a matter of justice.

The paper comprises an initial attempt to outline and broadly structure future research directions. As an agenda setting paper, it seeks to lay out broad claims about the terrain rather than set policy (which would clearly require wider input and consensus). Ultimately, our intent is to contribute to a “new proactive” research ethics agenda by drawing attention to domains where future bioethics research on priority-setting is needed and by providing preliminary ideas in relation to these areas. It is hoped that our initial attempt to define these issues will be met with critical attention and spur future investigations by ethicists, researchers, and policymakers.

## Main text

### Scope

In any discussion about the ethics of global health research priority-setting, the scope of the priorities to be set must first be clarified. Three *dimensions* can be identified along which the scope of priority-setting decisions might vary: the geographic region or entity to which the decisions apply, their macro or micro level focus (categories of research versus specific research questions), and the sources of research funding to which resultant priorities will apply. Any particular priority-setting exercise may be resolved into these dimensions.

The first dimension might be understood to capture the level at which priority-setting is undertaken. Workshop participants repeatedly emphasised that global health research priority-setting can occur at multiple, distinct levels that encompass global and national levels but also include funder, consortia, and institutional levels. At the national level, priorities are generally set for the health research that is supported within a particular country. It entails LMICs and high-income countries alike setting their own domestic health research priorities as well as high-income countries including health problems primarily experienced in LMICs in their priorities. Depending on the structures and health concerns within a given country, this priority-setting might stipulate fine-grained or broad brush research priorities. At the global level, research priorities are set that apply across countries worldwide and/or across regions and there is potential for many more actors to be involved in the decision-making process. The global health landscape has expanded tremendously over the past two decades, with many new actors emerging such as global health organisations (e.g. GAVI, Global Fund), philanthropic foundations (e.g. Gates Foundation), and product development partnerships (e.g. Drugs for Neglected Tropical Diseases) [31]. These priorities are, therefore, likely to be broad ranging and, in principle, based on global or regional trends and needs. Global health research consortia (alliances of universities, research institutions, and other organisations) often set priorities that are meant to guide the research projects undertaken by their member institutions. These priorities are likely to be driven in part by the concerns, interests, and goals of a given consortium and its members. How a particular donor sets priorities for the global health research it funds will only apply to eligible applicants and recipients and is likely to be driven by its broad institutional goals and interests. Finally, research institutions set priorities that apply to their internal funding schemes, which are typically open to their staff.

The second dimension refers to the intended breadth or specificity of the outputs of global health research priority-setting. Depending on whether broad or narrow priorities are sought, priority-setting may involve making macro-level decisions about broad areas of health research or micro-level decisions about specific health research questions. As affirmed in the Council on Health Research for Development’s *Manual for Health Research Priority-setting*, there must be follow-up action after identifying broad priority areas, which includes translating them into specific research questions [30]. For example, a ‘macro’ level decision could be: should HIV prevention research be prioritised over research on family violence prevention? A ‘micro’ level decision related to the priority area of HIV prevention research might then be: should a research question about the efficacy of a new PrEP regimen be ranked higher than a research question about the effectiveness of an intervention targeting the structural causes of HIV? Setting priority areas of health research seems to potentially be the remit of global, national, and donors’ priority-setting processes, whereas deciding upon specific research questions under those areas might be more applicable to consortia and research institutions.

Beyond identifying research priority areas and questions, priority-setting also encompasses applying those priorities (i.e. resource allocation decisions). A ‘macro’ level decision could be: should we create a grant program for biomedical research on neglected tropical diseases or a grant program for social determinants research on non-communicable diseases? A ‘micro’ level decision (within a grant program) could be: should a trial testing the effectiveness of a HIV vaccine candidate be funded over a trial testing the effectiveness of a specific sexual health education service? Applying priorities seems to be the remit of donors and research institutions, as they are the bodies that primarily oversee and implement grants programs.

The third dimension acknowledges that global health research is typically funded by multiple sectors (public, private for-profit, philanthropic) with distinct aims and accountabilities. At the national (and perhaps global level), a decision might be made (as part of the priority-setting process) about which research priorities should attract public sector funding and which should be left to the market or to philanthropies. Perhaps public funding should be used to support global health research on issues that market forces or charities do not. It might be that a particularly strong charity presence means that public funds should be redirected elsewhere. Alternatively, it might be important to manage or shape the incentives for private for-profit funders and/or philanthropic funders by designating priorities for them to address. Legal or regulatory instruments might be used in ways that can shape these decisions. The European orphan drug legislation is an example of this kind of incentive management. It provides commercial protection by allowing companies to hold patents for an extended period of time, which is intended to make investing in orphan drugs more commercially appealing to the private for-profit sector.

Given these dimensions, there is a pressing need for academic ethics research to investigate what relationship should exist between priority-setting at not only global and national levels but also funder, consortia, and institutional levels. As a starting point, workshop speakers proposed a bottom-up approach to global health research priority-setting may be ethically appropriate in a range of contexts as opposed to the top-down approaches often employed. This concept was supported by many (but not all) workshop participants. Bottom-up meant an approach where institutional priorities (informed by an “evidence of need”^2^ generated through community engagement) direct national priorities, which, in turn, direct global priorities. A normative basis for this claim might be found in accounts of health and social justice, using concepts of shared health governance and cognitive justice. Shared health governance calls for shared decision-making with meaningful participation of a wide range of health-related stakeholders, rather than top-down and hierarchical decision-making [39]. Knowledge democracy and cognitive justice recognise the right of different systems of knowledge to exist as part of dialogue and debate [43, 45]. They draw attention to inequalities in the knowledge that is valued and produced in today’s world (e.g. “Northern” epistemologies over “Southern” epistemologies^3^ and expert knowledge over local and indigenous ways of knowing) and call for such inequalities to be rectified [20].

Even so, significant questions remain about whether and when a bottom-up approach is ethically preferable to a top-down approach. Arguably funders’ research agendas might be usefully informed by national priorities and locally-driven institutional priorities. The range and complexity of these decisions might mean that alternative approaches are appropriate instead. The context-sensitive ethical justification for bottom-up or other approaches to connecting the different levels of priority-setting in global health research should be explored further. Additionally, global health research priority-setting processes of varying scope (i.e. differing across the three dimensions) will generate both overlapping and distinct ethical considerations relative to one another. Conceptual and empirical work is needed to determine what ethical considerations arise and how they should be addressed given the scope of research priority-setting undertaken.

### Values and substantive considerations

A key overarching ethical question is what values and substantive requirements should guide or underpin global health research priority-setting. As noted by Nuyens ([29], p. 320-1) “setting priorities means making choices, and those choices must refer to defined underlying values.” Health justice has been identified as one such guiding value for health research priority-setting in the literature [29, 36]. To ensure such processes generate outputs that advance health justice, it is important to identify substantive ethical criteria to guide them. But what substantive criteria should be applied at the different levels? Several criteria, which may be necessary to apply in global health research priority-setting, were suggested by workshop participants: need, magnitude of benefits, equity, the needs of vulnerable and disadvantaged groups, cost-effectiveness of research, cost-effectiveness of proposed interventions, and likelihood of research success.

In global health research priority-setting, the temptation is to think that research targeting conditions with the highest health burden should be prioritised as a matter of justice. Instead, workshop participants recognised that the decision is much more complex than that and identified, *need*, rather than burden of disease, as a possible criterion for global health research priority-setting. The two concepts are not identical; burden of disease considerations alone are insufficient to establish that research addresses a health need. What comprises a health need depends in part on the concept of health and the causal explanation of what determines health being used.^4^ Important conceptual and empirical research questions thus remain about what constitutes a health need and how we should understand the relationship between the health needs of individuals, the health needs of groups or larger populations, and the burden of disease. Another topic to investigate is how to prioritise research that addresses different health needs such as longer length of life versus better quality of life, severe diseases versus prevalent diseases, or child health versus ageing. How these particular health needs ought to be balanced and traded against each other will vary according to different accounts of health and social justice. For example, Powers and Faden’s theory of social justice emphasises protecting health early in the life course [33]. It would support using criteria or weighting criteria in ways that favour research priorities focused on children’s health needs.

The *magnitude of the benefits* (if the research hypothesis is successfully proven), *equity*, and *the needs of vulnerable and disadvantaged groups* (as distinct from needs generally) were also proposed as potential substantive criteria by workshop participants. The former criterion entails considering the size and type of expected benefits of the research for the host population. The latter two criteria encompass assessing whether the research has potential to generate evidence that will help reduce unjust health disparities and whether the research has potential to address vulnerable and disadvantaged groups’ most pressing health needs. Tensions will likely arise between, for example, consideration of magnitude of benefits and equity, and how to navigate them needs to be investigated.

The *cost-effectiveness of research* and the *cost-effectiveness of proposed interventions* are additional potentially relevant criteria. The former comprises considering is the research itself a good use of resources and the latter means considering is the intervention good value for money (compared to existing interventions). As part of further specifying these criteria, how cost-effectiveness should be defined will be important to explore. Questions remain about what constitutes cost-effectiveness in global health research contexts. The assumptions and analytical tools used in cost-effectiveness analysis reflect certain values and setting-specific valuations, and they can violate ethical principles like equity [40]. It is also noted that trade-offs between the two cost-effectiveness considerations might occur during micro-level application of priorities. For example, should a donor support research proposal 1—a large clinical trial with a moderate chance of success that, if successful, will produce intervention A, which will be affordable and effective on a large scale. Or should the donor invest in research proposal 2—a series of comparatively inexpensive, small-scale studies that are likely to succeed and will produce intervention B, which is expensive and treats a relatively rare, localised health problem.

The *likelihood of the research being successful* may be another pertinent criterion and would encompass consideration of, for example, the background evidence supporting the research, its novelty, and the time it will take to yield results. Yet workshop participants noted a tension exists between prioritising research with high versus lower degrees of uncertainty in outcomes (e.g. basic science to support drug discovery versus health systems research testing the efficacy of a proven delivery model in a new location) since research with a high degree of uncertainty might bear fruit in the long-term. Recognition of the potential for this criterion to bias priority-setting against basic science research might be appropriately reflected in its weighting relative to other criteria.

Further bioethics scholarship is needed to determine the ways in which the proposed criteria are best specified to promote health justice and how their specification may vary between priority-setting at different levels. It should also explore what weights might be assigned to the various criteria. Scholars have argued that the goal of health research priority-setting is to define a portfolio that has the greatest impact on the health of the worst-off or disadvantaged [4, 36]. There was strong support for this view at the workshop, where it was suggested that helping improve the health of the worst-off in a country, region, or even globally ought to be an over-riding ethical value for setting research priorities. This would entail weighting substantive criteria related to equity more heavily in health research priority-setting than those related to utility and cost-effectiveness. Certain types of research questions have been identified as more likely to benefit people who are disadvantaged: 1) research questions to develop more affordable, less technology-dependent versions of existing medical products; 2) implementation research questions to ensure effective interventions are integrated into health systems; and 3) health systems research questions more broadly [4]. Assigning an equity criterion a high weight would likely favour identifying such research questions as priorities.

Finally, bioethics scholarship is needed to determine what *other* values should also or alternatively be pursued through global health research priority-setting and what upholding those additional values entails (in terms of substantive criteria) at the different levels of priority-setting. It is important to observe that health research priority-setting decisions across levels may well involve differing sets of values and so the set of relevant criteria to be considered at each level may vary. Exploring the trade-offs between different values and their associated criteria will also be critical.

### Procedural considerations

Theories of justice generally call for using deliberative processes and norms to achieve fair or just priority-setting [18, 49]. Drawing on these theories, the well-known ‘Accountability for Reasonableness’ (A4R) framework was developed as a guide for achieving fair priority-setting processes [13, 14]. The rationale was that, in the absence of consensus about how specific values should guide priority-setting decisions, it is essential to put in place procedures that will ensure the fairness and legitimacy of those decisions [42].

With the A4R approach in mind, three *procedural* considerations that can promote fairness and justice are identified that apply to each level (global, national, funder, consortia, institutional) of priority-setting:

- What is the right process for making decisions about global health research priorities?
- Who should initiate and lead global health research priority-setting?
- Who should participate in global health research priority-setting and how should they participate?

Specific sub-questions for exploration are highlighted below within each of these broader areas. Some of these sub-questions may apply to all levels of priority-setting whereas others may only be relevant to a particular level(s).

#### What is the right process for making decisions about global health research priorities?

Workshop participants affirmed, at a minimum, the right process should be a fair process; procedural requirements for a *fair* health research priority-setting process then need to be identified. This may demand requirements corresponding to norms of deliberative decision-making such as transparency, reciprocity, non-coercion, qualitative equality, and accountability amongst others [18, 21]. It is possible that A4R requirements (relevance, publicity, appeals/revision, and enforcement) might be adapted for global health research priority-setting as part of future conceptual work. However, it will be important to take account of existing criticisms and limitations of using the A4R approach when doing so. For example, Gibson, Martin, and Singer [15] have proposed that, beyond A4R, a requirement for promoting qualitative equality is also vital to ensuring fairness in decision-making in contexts of power disparities. Upholding such a requirement entails, first, identifying where power differences exist between participants in a given decision-making process and, second, developing and implementing strategies to minimise those power differences.

Sub-questions for exploration then include: (i) what deliberative norms should apply in global health research priority-setting and how should procedural requirements reflecting them be specified? (ii) are additional procedural requirements ethically required to achieve fairness and justice? A local ownership requirement may be needed and could perhaps be reflected in how the next two procedural considerations under discussion are specified. A requirement for using “interpretive” priority-setting methods rather than technical, disease-driven methods may apply where global health research priority-setting encompasses selecting both biomedical and non-biomedical research priorities or only non-biomedical research priorities. As previously noted, the use of “interpretive” priority-setting methods may be essential as a matter of health justice because types of non-biomedical research are needed to improve the health of the most disadvantaged. Requirements for the way in which a global health research priority-setting process is set up and justified (and by whom) may also be important because they can help establish the ethical legitimacy of the resultant priorities.

#### Who should initiate and lead global health research priority-setting?

Workshop participants identified leadership of priority-setting processes and determining who should ideally initiate such processes as an important ethical consideration. The actors or institutions who assume or are given the responsibility to undertake a global health research priority-setting exercise gain considerable power, as they make a number of decisions that shape how the priority-setting process is conducted. For instance, they determine who is included in the process and whether it is structured in a way that promotes different stakeholders having an equal opportunity to participate. They may even have control over the substantive criteria used to set research priorities.

A workshop speaker noted that empirical evidence shows nearly half of health research priority-setting processes in LMICs are initiated by international organisations, with only a third initiated by LMIC governments and an even lower proportion (15%) commenced by LMIC researchers [25]. Where national or institutional research priority-setting is not led by in-country actors, it generates ethical concern that there is a lack of local ownership of the process. This reflects concern over whose priorities are ultimately reflected in the outputs of priority-setting—namely, local or external actors—based on who “owns” the process. Given that approaches like A4R rest on the idea that justice requires all parties to have (and be seen to have) an equal opportunity in deliberations, the potential for these kinds of biases are ethically significant.

Sub-questions for exploration then might include for example: (i) which actors, donors, or institutions should lead or control global health research priority-setting processes at the different levels? (ii) what is the role of the international scientific community in priority-setting processes? At the national level, priority-setting could be led by the ministry of health, the national research council, or particular prominent research institutions. According to workshop participants, the appropriate actor to initiate priority-setting may be national health research councils because they are responsible for overseeing research in many countries. Yet this may be difficult to implement in practice since some countries do not have bodies that coordinate health research and others have more than one. Moreover, government support and public trust in such bodies may be quite limited. In such cases, who should lead national research priority-setting? At the global level, it is even less clear which type of actor(s) and/or institution(s) should be in charge of leading and initiating global health research priority-setting.

#### Who should participate in global health research priority-setting and how should they participate?

Relevant ethical considerations about participation in global health research priority-setting that apply at each level are: who should be involved and how should they be involved. Workshop attendees suggested participants could potentially include national and sub-national stakeholders spanning the weak and powerful, the organised and unorganised, research experts, policymakers, healthcare providers, citizens, and independents (i.e. those who are not stakeholders per se but can ensure that the process functions fairly). An account of deep inclusion in health research priority-setting argues that involving research-producers, research-users, and research-beneficiaries is necessary but not sufficient. Achieving deep inclusion means ensuring that participants also span a wide spectrum of relevant roles and demographics within those three categories [36]. That account and the A4R framework further emphasise that disadvantaged and vulnerable groups must participate in health-related priority-setting as a matter of fairness and justice [17, 36]. How to ensure the involvement of the disadvantaged and less powerful was raised as a key concern by workshop participants.

A key sub-question in this context might then be: What is required to achieve fully inclusive global health research priority-setting at the different levels? A comprehensive account would speak to what types of actors must participate and across what demographics, what mode of participation should be afforded to different actors, what phases of the priority-setting process they should participate in, and how the disadvantaged and vulnerable should participate. A variety of modes of participation exist, with some more “active, deliberative, and influential” than others ([11], p. xxvii). Sherry Arnstein and others distinguish between lay control, partnership, and consultation [1, 8, 27, 38]. Lay control means citizens are solely responsible for decision-making with (at most) consultative input from experts. Collaboration or partnership involves shared decision-making between experts and citizens [38]. Consultation is characterised by citizens being invited to give their input but having no assurance that it will be used by those who decide [1]. It may entail all or some of the following: proposal-sharing, information-giving, and/or providing feedback^5^ [9]. A number of phases in “interpretive” health research priority-setting have also been identified: planning the priority-setting process, identifying research topics and ranking criteria, and setting health research priorities. It has been argued that earlier entry into the process is associated with deeper inclusion [36].

Other related sub-questions to explore include: (ii) what role should LMIC actors play in priority-setting at the global level? (iii) should external actors be allowed to participate in national and institutional health research priority-setting? Currently, LMIC actors (e.g. general public, researchers, policymakers) typically play a limited part in setting what are meant to be global priorities [29]. Their taking on a bigger role may be warranted as a matter of fairness and justice. External donors often exert undue influence on national and institutional health research priority-setting in LMICs because they provide the majority of research funding in LMICs [16]. As an example, during the course of one year, a Faculty within a South African research institution received a total of $232,000 USD in funding from within its country compared to $10,786,000 USD from the United States’ National Institutes of Health. When the relative proportions of health research funding from outside and within the country are compared, it demonstrates the extent to which international funding and the priorities set by external donors can influence what research is prioritised and pursued locally. A primary role for LMIC actors may instead be generally warranted in their national and institutional research priority-setting as a matter of justice. Accounts from political philosophy like shared health governance would support such an approach [35].

Even if a country-based approach is warranted, it is also necessary to consider under what circumstances the “autonomy” of sub-national and national stakeholders can permissibly be “influenced” by the involvement of external actors in national or institutional priority-setting. Workshop participants suggested that the participation of external actors may be ethically permissible in some contexts such as where countries have corrupt governments or where certain requirements for the legitimacy of the priority-setting process are not met. What those “legitimacy” requirements are and who determines if they have been met are additional questions for exploration.

A final set of sub-questions relates to the involvement of communities: (iv) what is the nature and purpose of community engagement in global health research priority-setting? (v) is community engagement necessary at each level of priority-setting? Community engagement using various mechanisms and with different understandings of “community” can, where appropriate, be built into, be prior to, or sit alongside global health research priority-setting processes. Workshop speakers affirmed the potential for community engagement to play a specific and key role in global health research priority-setting—namely, capturing an “evidence of need” to inform the process. For example, they proposed community engagement could inform national priority-setting via institutional priority-setting; different research institutions could be responsible for engaging with their surrounding communities, including the disadvantaged within them, and incorporating their “evidence of need” into institutional research priorities. Accessing the voices of more disadvantaged community members would be important to making their health needs visible in research priorities. This more local or ground-level evidence could then more thoroughly inform decisions about national and global priorities. Where robust community engagement has occurred at the institutional level and its outputs are used to inform higher level priority-setting, it may then not be necessary as part of national or global processes.

## Conclusions

The ethical issues involved in setting global health research priorities are complex and under-researched. In this paper, we identify and frame a research agenda for these complex ethical issues so that they can be studied further (Table 1). Three sets of ethics questions emerge. First, there are pressing ethical issues about the interaction and relationship between the various scope distinctions within global health research priority-setting. Under what circumstances and in what way should institutional or national research priorities influence, or be influenced by, global priorities? Workshop participants proposed a case could be made for bottom-up approaches in some contexts. This idea received support from many (but not all) workshop attendees.

**Table 1:** A research agenda for the ethics of global health research priority-setting

Domain of Priority-setting	Research Questions	Indicative Sub-questions
Scope	What relationship should exist between global health research priority-setting processes at global, national, funder, consortia, and institutional levels?	• When is a bottom-up approach ethically preferable to a top-down approach to connect the different levels of priority-setting?
	What ethical considerations arise and how should they be addressed for global health research priority-setting processes of varying scope?	• At the national level, which research priorities should attract public sector funding and which should be left to the market or philanthropies?
Substantive	To advance the value of health justice, what substantive criteria should guide global health research priority-setting at different levels?	• What weights should be assigned to the substantive criteria that guide global health research priority-setting? • What tensions arise between substantive criteria and how can they be navigated?
	What is the best way to specify the substantive criteria that promote health justice and should their specification vary between priority-setting at different levels?	• How should we understand and measure health needs in the global health context? • How should cost-effectiveness be defined in global health research contexts?
	What other values should be pursued through global health research priority-setting at different levels and how do they relate to health justice?	• To advance those other values, what substantive criteria should guide global health research priority-setting? • What tensions exist between these criteria and those that advance health justice?
Procedural	What is the right process for making decisions about global health research priorities at the different levels?	• What deliberative norms should apply in global health research priority-setting and how should procedural requirements reflecting them be specified? • Are additional procedural requirements ethically required to achieve fairness and justice?
	Who should initiate and lead global health research priority-setting at the different levels?	• Which actors, donors or institutions should lead or control global health research priority-setting processes? • What is the role of the international scientific community in priority-setting processes?
	Who should participate in global health research priority-setting and how should they participate at the different levels?	• What is required to achieve fully inclusive global health research priority-setting? • Should external actors be allowed to participate in national and institutional global health research priority-setting? • Is community engagement necessary at each level of priority-setting? • What is the nature and purpose of community engagement in global health research priority-setting?

The second set of ethics issues involves being clear about the values and associated substantive requirements that are at stake in global health research priority-setting. What is the range of values and relevant substantive criteria that should be taken into account when prioritising global health research at each of the levels? What is the best way to specify the various substantive criteria? There was strong support at the workshop for the view that helping improve the health of the worst-off in a country, region, or even globally ought to be an over-riding ethical value for setting global health research priorities.

The third set of ethics questions concerns the nature of the processes that should be used to ensure fair and just decision-making about research priorities at each level. What is the right process for making decisions about global health research priorities? Who should initiate and lead global health research priority-setting? Who should participate in global health research priority-setting and how should they participate?

We take these three areas of investigation to set an important agenda for academic ethics research. Both normative and empirical ethics methods can usefully be employed to explore them. We emphasise the need for more work, debates, and discussions amongst researchers, ethicists, and policymakers in all three areas and hope this call to action will stimulate an active global discourse on the ethics of global health research priority-setting. We further note that, while this paper has focused on global health research, our arguments and research agenda may be relevant to research priority-setting more generally.

## Abbreviations

A4R: Accountability for Reasonableness
GAVI: Global Alliance for Vaccines and Immunization
LMIC: Low and Middle-Income Country
USD: United States Dollars

## Footnotes

1 A point of convergence among multiple theories of social justice is that it is a priority and duty of justice to avert and alleviate disadvantage [3].

2 This term was used by workshop speaker Professor James V. Lavery (Emory University) in his presentation.

3 Here, “the global South is not a geographical concept, even though the great majority of its populations live in countries of the Southern hemisphere. The South is rather a metaphor for the human suffering caused by capitalism and colonialism on the global level… It is a South that also exists in the geographic North (Europe and North America), in the form of excluded, silenced and marginalised populations, such as undocumented immigrants, the unemployed, ethnic or religious minorities, and victims of sexism, homophobia, racism and islamophobia” ([41], p. 18–19).

4 Depending on what concept of health is used, needs may be understood to comprise deficits in physical and mental well-being (from an optimal or decent level) or they may be defined more broadly. Venkatapuram’s account of health justice, for instance, defines health as individuals’ ability to achieve ten central human capabilities: life; bodily health; bodily integrity; senses, imagination, and thought; emotions; practical reason; affiliation; other species; play; and political and material control over one’s environment [44]. Causal models of health identify different bases of individuals’ ability to be healthy: biological, agency, physical, and/or social. Depending on what causal bases are recognised as pertinent, needs may be defined as what is required to generate improvements in: individuals’ biological capacity to achieve health, individuals’ ability to act as agents of their own health and achieve health goals they value, enabling physical environmental conditions, and/or social and material structures that promote health.

5 In proposal-sharing, citizens provide their recommendations or suggestions for what they would decide if they had the power to make certain decisions. They offer their “demands” for new research priorities, topics, or questions to be investigated by those with decision-making power [9]. In information-giving, citizens provide information on topics that have been selected by those who created the decision-making space and/or by themselves. Their information may or may not be used. Finally, providing feedback means that citizens share their opinions on the outputs or other aspects of a decision-making process. They give judgements about the relevance of research priorities set by others [9].
